# Case Report: Successful management of refractory SAPHO syndrome with guselkumab-upadacitinib combination

**DOI:** 10.3389/fimmu.2026.1745464

**Published:** 2026-02-20

**Authors:** Di Jin, Ming Yuan Xu, Xiao Li Wang, Ruo Qi Wang, Jun Ting Tang, Ye Qiang Liu

**Affiliations:** 1 Department of Dermatopathology, Shanghai Skin Disease Hospital, Tongji University School of Medicine, Shanghai, China; 2 Department of Dermatopathology, Shuguang Hospital Affiliated to Shanghai University of Traditional Chinese Medicine, Shanghai, China; 3 Department of Graduate School, Tongji University School of Medicine, Shanghai, China; 4 Department of Dermatopathology, Shanghai Children’s Hospital, Shanghai, China

**Keywords:** guselkumab, IL-23 inhibitor, JAK inhibitor, SAPHO syndrome, upadacitinib

## Abstract

SAPHO syndrome is a rare chronic aseptic inflammatory disorder characterized by the concurrence of osteoarticular inflammation and cutaneous lesions. The intricate mechanism network, coupled with issues such as paradoxical reactions, often results in suboptimal therapeutic outcomes. To the best of our knowledge, this is the first report of refractory SAPHO syndrome achieving clinical remission with the combination of guselkumab (an IL-23 inhibitor) and upadacitinib (a JAK inhibitor). A 38-year-old male with refractory SAPHO experienced complete pain resolution within 2 weeks and significant improvement in cutaneous lesions by 4 weeks post-treatment, with a favorable safety profile observed throughout the follow-up period. We further analyzed the underlying inflammatory mechanisms to provide a therapeutic clues for the management of similar refractory cases.

## 1 Introduction

Synovitis-acne-pustulosis-hyperostosis-osteitis (SAPHO) syndrome is a rare chronic aseptic inflammatory disorder characterized by concurrent osteoarticular inflammation (e.g., chest/back pain, joint tenderness) and cutaneous lesions (e.g., palmoplantar pustules, acne nodules) (1). Its main pathogenesis involves the dysregulation of multiple inflammatory pathways (e.g., IL-23/Th17 axis (2), TNF-α pathway (3). Previously, NSAIDs, steroids, antibiotics, bisphosphonates, and conventional DMARDs (cDMARDs) were routinely used in the treatment of SAPHO syndrome. Nowadays, an increasing number of patients are treated with biological agents, including inhibitors targeting IL-12/23, IL-17, TNF-α, IL-1β, IL-6, IL-8, and JAK. However, the therapeutic efficacy is unsatisfactory in refractory patients, such as those with paradoxical reactions, who may require combined therapy with two or more drugs. Therefore, it is of great importance to select biological agents based on individual patient conditions. Herein, we report a case of SAPHO who had poor responses to traditional medications, and subsequent treatment with biological agents such as TNF-α and IL-17 inhibitors also failed to achieve satisfactory efficacy (4). Eventually, the patient achieved significant improvement after administration of guselkumab (an IL-23 inhibitor) and upadacitinib (a JAK inhibitor). This case describes their successful use and analyzes underlying mechanisms.

## 2 Case report

**Figure 1:**
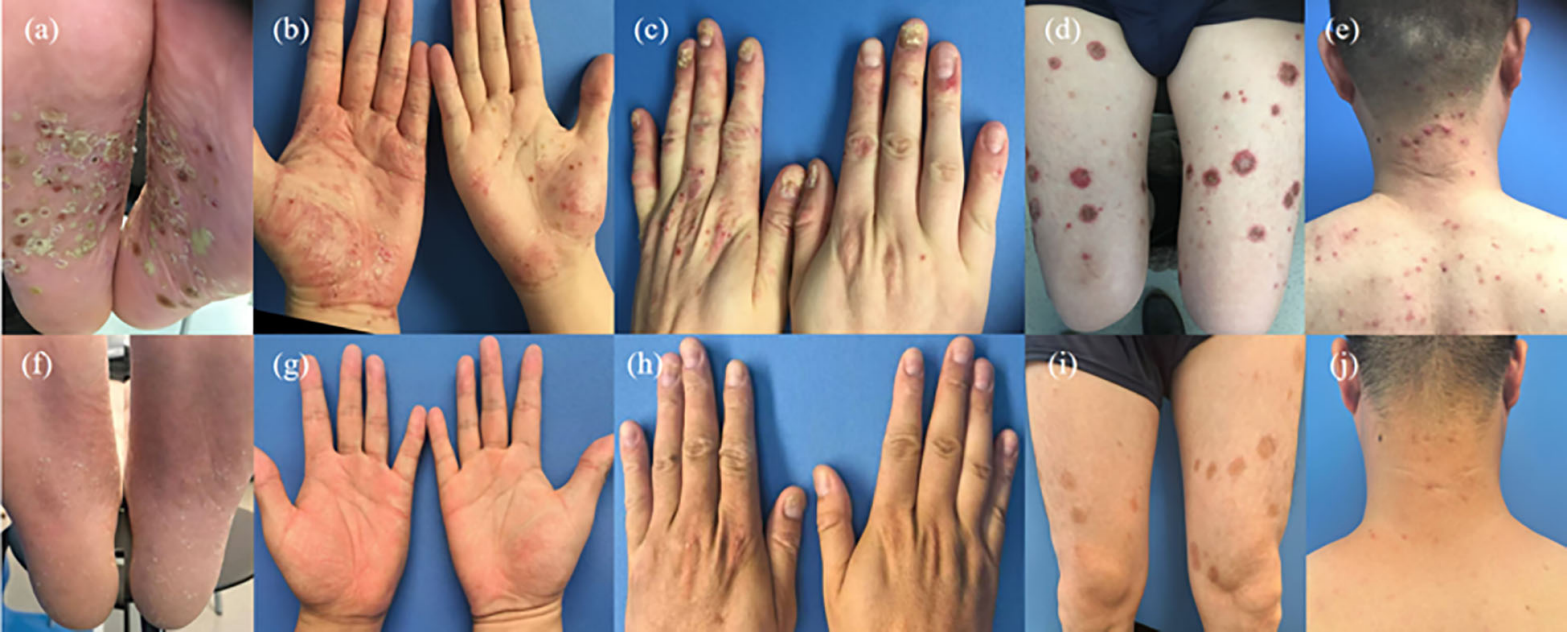
**(a)** Clinical changes. Pre-treatment plantar pustules; **(b)** Pre-treatment palmar pustules; **(c)** Pre-treatment onychomatrix opacity; **(d)** Pre-treatment limb erythematous scales; **(e)** Pre-treatment head/neck acne; **(f)** 24-week normal soles; **(g)** 24-week normal palms; **(h)** 24-week normal nails; **(i)** 24-week limb mild pigmentation; **(j)** 24-week normal head/neck skin.

**Figure 2:**
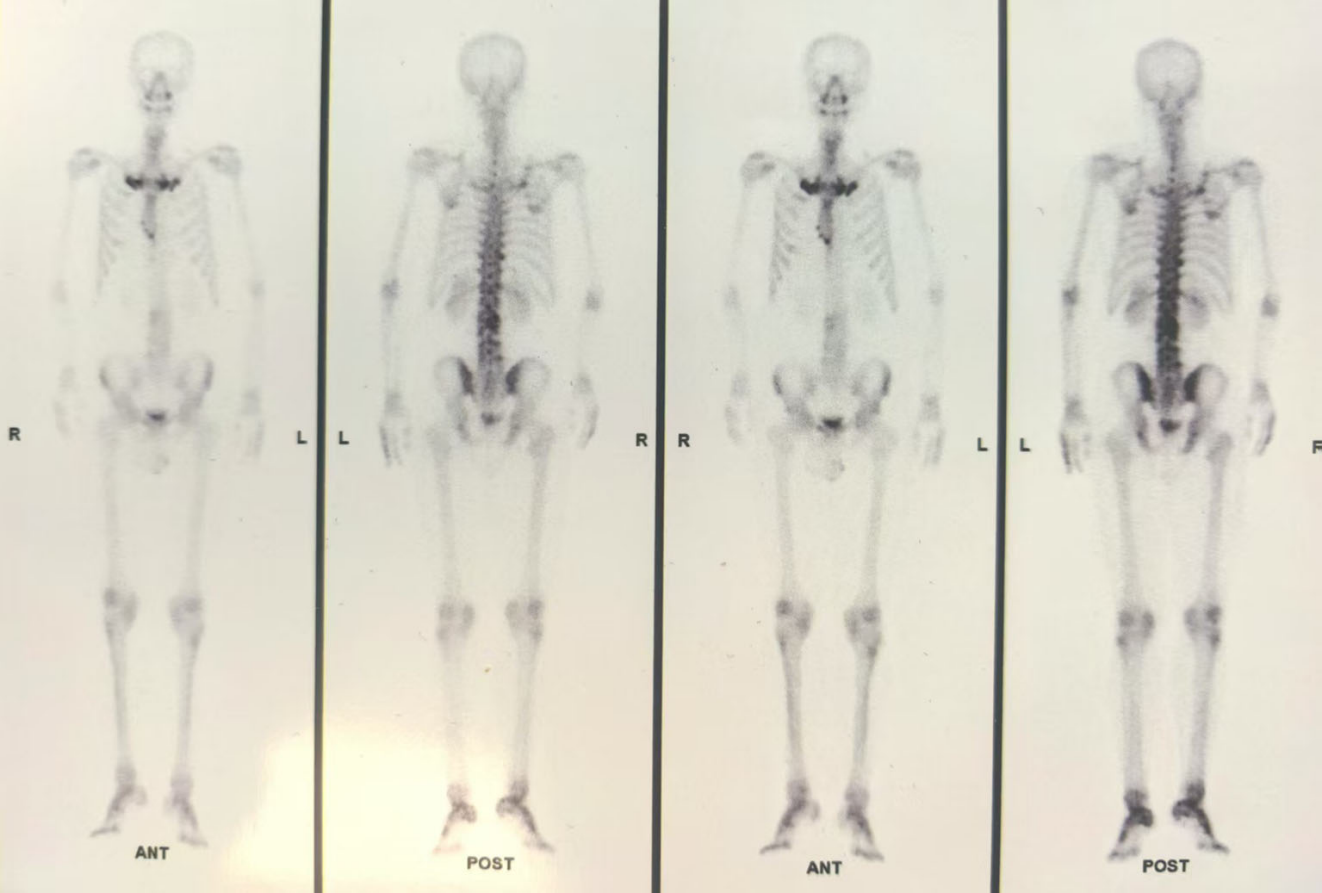
Bone scintigraphy. Pre-treatment increased radioactive uptake (tomographic slice thickness: 5 mm). Bone scan findings before treatment indicated typical “bull head sign”, osteomyelitis and Synovitis.

A 38-year-old Chinese male had 14 years of recurrent palmoplantar diffuse erythema with pustules and 7 months of chest/back pain accompanied by morning stiffness[Visual Analog Scale (VAS) score: 9]. Prior to referral, he had received tripterygium glycosides tablets, which provided temporary symptom relief but was followed by exacerbation shortly after drug discontinuation. In September 2024, he was diagnosed with SAPHO syndrome at an external hospital. Treated with methotrexate (15 mg/week) combined with etoricoxib (60 mg/day), plus three ineffective targeted therapies: first, etanercept (50 mg/week,1 month), caused new-onset facial acne and scaly plaques on the legs, with no relief of chest/back pain; subsequently, secukinumab(150 mg) leading to progressive exacerbation of scaly plaques on the legs and pustules on both palms; finally, upadacitinib(30 mg/day, 1 month) decreased pain of sacroiliitis (VAS 4) but cutaneous symptoms deteriorated, leading to referral to our hospital in February 2025. And she then underwent a skin biopsy of her right hand and other examinations (results are as follows). Physical examination showed palmoplantar erythema with dense pustules (partially fused into pustular lakes, **Figures 1a, b**), approximately 30 acne nodules on head and neck (**Figure 1e**), widespread oyster shell-like erythematosquamous plaques, distributed on the limbs and trunk, mainly on the extensor side (30% body surface area, Auspitz sign positive, **Figures 1c, d**), 2 missing nail plates with periungual erythema and pustules; chest, back and sternoclavicular tenderness and 10% movement limitation (VAS 4). His mother only had palmoplantar pustulosis and has been using topical medications for a long time to treat the skin rash.

**Figure 3:**
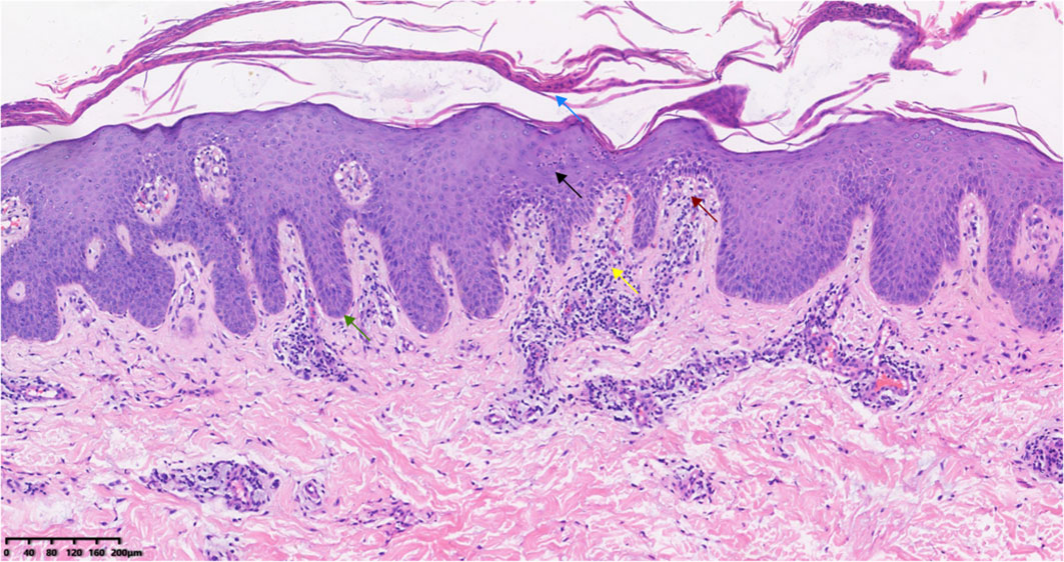
Pathological manifestations: focal parakeratosis (blue arrow), downward elongation of rete ridges(green arrow), intraepidermal accumulation of neutrophils forming Pautrier microabscesses(black arrow), dilated capillaries between the rete ridges(red arrow), and dermal infiltration of inflammatory cells including histiocytes and lymphocytes(yellow arrow)(HE, 10X).

Laboratory tests: elevated complement C3 (1.630 g/L), C4 (0.454 g/L), erythrocyte sedimentation rate (36 mm/h), c(652.84 pg/ml); HLA-B27 and T-SPOT.TB negative. Imaging: lumbar MRI showed abnormal signals in the T12, L2-L5 vertebrae, which suggested sacroiliitis; bone scintigraphy (ECT) showed increased radioactive uptake in the bilateral 1st ribs and right 2nd anterior rib, which suggested hyperostosis “bull’s head sign”; Increased radiotracer uptake is noted in the second and third lumbar vertebrae and lateral malleolus, which suggested osteomyelitis and Synovitis (**Figure 2**). Pathological findings (February 2025) from biopsy of her right hand revealed focal parakeratosis, downward elongation of the rete ridges, microabscesses with neutrophilic aggregation, telangiectasia, as well as perivascular and superficial dermal infiltration of lymphocytes and histiocytes, which are highly consistent with the diagnosis of palmoplantar pustulosis (**Figure 3**).

The patient was diagnosed with SAPHO syndrome according to the criteria established by Kahn et al. (2003), fulfilling the first of the four criteria: Bone-joint involvement associated with palmoplantar pustulosis and psoriasis vulgaris. Additionally, Infectious osteitis and tumoral conditions of the bone were excluded by means of MRI, ECT, blood routine tests and other relevant examinations. Owing to unsatisfactory responses to multiple cycles of standardized conventional treatment, the diagnosis of refractory SAPHO syndrome was finalized in reference to pertinent literature (5).

Given that the patient was diagnosed with a refractory disease and presented with complicated clinical conditions, a combination therapy with two medications was administered. The patient was treated with guselkumab (100 mg subcutaneous injection at Weeks 0, 4, 8, then q8w) and upadacitinib (30 mg/day orally) and topical halometasone ointment. Regularly recheck the blood routine, liver and kidney functions, and chest computed tomography (CT). Follow-up: 2 weeks (pain resolved, VAS 0; morning stiffness disappeared); 4 weeks (numbers of right palmoplantar pustules decreased 50→10, PASI decreased 24.3→8.1); 24 weeks (cutaneous symptoms essentially resolved, **Figures 1f-j**); 30 weeks (malassezia folliculitis cured with topical selenium disulfide lotion) (**Table 1**).

**Table 1:** Patient follow-up results.

Follow-up time	Chest/back pain VAS	Right palmoplantar pustules(numbers)	PASI score	Nail lesion status	Adverse reactions
First Visit	4	50 (**Figures 1a, b**)	24.3 (**Figure 1d**)	Thickened/deformed, periungual redness/pustules (**Figure 1c**)	None
2 Weeks after treatment	0	80	21.6	Thickening stopped	None
4 Weeks after treatment	0	10	8.1	Thinned, redness subsided	None
24 Weeks after treatment	0	No new pustules (**Figures 1f, g**)	6.0 (**Figure 1i**)	Regeneration, normal periungual area(**Figure 1h**)	None
30 Weeks after treatment	0	No new pustules	1.8	Regeneration, normal periungual area	Scattered follicular papules on chest/back/upper limbs

a Transient increase of pustules at 2 weeks after treatment considered pre-drug temporary inflammation release before drug onset, which subsided rapidly thereafter.

## 3 Discussion

SAPHO syndrome is a rare chronic aseptic inflammatory disorder. Its potential etiologies include immune dysfunction, infection (1, 6), and genetic susceptibility (7), characterized by synovitis, acne, pustulosis, hyperostosis, and osteitis as core manifestations. The classic finding of ECT is the “bull’s head sign” (6). Serological tests may show elevated white blood cell count, erythrocyte sedimentation rate, and C-reactive protein level, while rheumatoid factor and antinuclear antibody are mostly negative. Skin and bone biopsies of SAPHO lack specificity; cutaneous histopathology reveals aseptic inflammation and pseudopustules formed by neutrophil infiltration, with negative bacterial cultures (8). The duality and superposition of cutaneous and osteoarticular lesions in this case underscore the complexity of differential diagnosis and the necessity of combined therapy.

In SAPHO syndrome, studies have demonstrated elevated expression of pro-inflammatory cytokines—including tumor necrosis factor α (TNF-α), interleukin-1β (IL-1β), IL-6, and IL-8—which collectively contribute to enhanced inflammatory responses (3). Concurrently, Key cytokines such as TNF-α, IL-1β, and IL-6 further act in synergy with signaling pathways such as JAK/STAT to activate synovial fibroblasts and osteoclasts, thereby driving synovitis, arthritis, and bone destruction (3).

**Figure 4:**
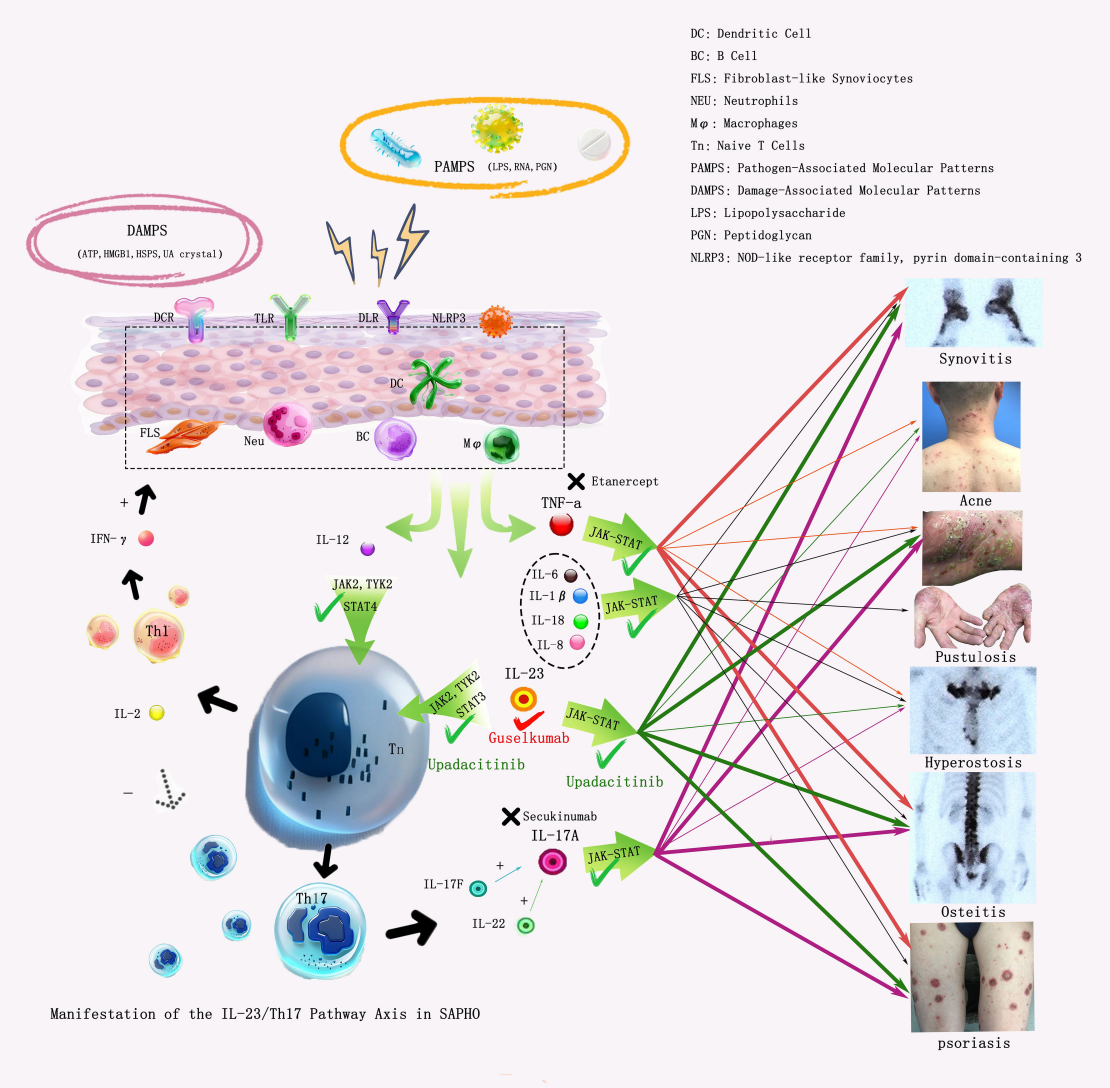
IL-23/Th17 Pathway Axis in SAPHO. When the skin is exposed to PAMPs or DAMPs, it activates cells such as dendritic cells (DCs), neutrophils (NEUs), B cells, macrophages, and fibroblasts via receptors including DCRs, TLRs, and NLRP3, which in turn secrete a variety of cytokines such as IL-12/23, TNF-α, IL-1β, IL-6, and IL-8. Among these, IL-12/23 activates T cells through multiple pathways including the JAK pathway, inducing their differentiation into Th17 and Th1 cells. Th17 cells then secrete IL-17, which acts on the body through the JAK pathway and other signaling cascades, thereby contributing to the development of palmoplantar pustulosis, psoriasis, and synovitis in SAPHO syndrome. In contrast, TNF-α are primarily involved in the pathogenesis of synovitis, psoriasis, and osteitis (11). IL-1β, IL-6, and IL-8 is secondary involved in the occurrence and development of Synovitis, acne, pustulosis, hyperostosis and osteitis.

IL-23, secreted by dendritic cells and macrophages, plays a central role in maintaining and expanding the Th17 cell population, which serves as the primary source of IL-17. IL-23 activates the JAK/STAT signaling pathway, thereby promoting Th17 cell differentiation and survival (8–10). In SAPHO syndrome, dysregulation of the IL-23/IL-17 axis is closely associated with the characteristic cutaneous and osteoarticular manifestations observed in SAPHO syndrome (**Figure 4**).

Currently, there is no consensus on the management of SAPHO. Several studies have demonstrated significant efficacy of TNF-α antagonists in SAPHO treatment (11). Wang et al. reported that IL-17 inhibitors improved palmoplantar pustulosis and bone marrow edema (12). IL-1 antagonists (13), JAK inhibitors (14), and PDE-4 inhibitors (15)have also shown favorable therapeutic effects, particularly in ameliorating osteoarticular and cutaneous symptoms.

Reports have shown that guselkumab, which targets the IL-23p19 subunit, acts on upstream IL-23 in the inflammatory pathway, inhibiting psoriasis-related inflammatory cascades induced by the IL-23/Th17 axis (16), and exhibits certain efficacy in the treatment of palmoplantar pustulosis (17). It also downregulates Th17 cell-mediated osteoclast activation, relieving osteoarticular inflammation (13). Additionally, risankizumab (an IL-23 inhibitor) has been reported to rapidly and sustainably alleviate SAPHO-related symptoms (18).A case of SAPHO successfully treated with ustekinumab (an IL-23 inhibitor) has also been reported (2).

The patient was treated with etanercept (TNF-α antagonist), secukinumab (IL-17 inhibitor), methotrexate (folate antagonist), and etoricoxib (NSAID). However, therapeutic response remained suboptimal due to: TNF-α inhibitors mainly act to alleviate symptoms of synovitis, psoriasis, and osteitis, while IL-17 inhibitors primarily improve palmoplantar pustulosis, psoriasis, and synovitis in SAPHO syndrome. Relevant studies have reported that TNF-α inhibitors may induce paradoxical reactions in the treatment of SAPHO, leading to the onset of psoriasis (19). Consequently, the administration of TNF-α inhibitors in this patient also resulted in exacerbated psoriasis like rashes, manifesting as a paradoxical reaction. Subsequent treatment with an IL-17A inhibitor yielded a suboptimal response, indicating the presence of this paradoxical reaction as well as the complexity of the patient’s condition. Therefore, a single early-line treatment regimen failed to relieve the patient’s symptoms. In contrast, following the initiation of upadacitinib, the patient experienced improvements in arthritis and pain relief, while cutaneous lesions remained refractory. Consequently, we opted for dual-targeted therapy with an IL-23 antagonist, as this combinatorial approach not only addresses multiple pathogenic pathways but also minimizes the risk of infection.

Upadacitinib (a JAK inhibitor) is widely used in the treatment of psoriatic arthritis and other conditions, with numerous reports documenting its efficacy in improving osteoarticular symptoms. A relevant study reported a case of refractory psoriasis and psoriatic arthritis successfully treated with the combination of guselkumab and upadacitinib (20). Similar to our case monotherapy may not be sufficient for the complete control of the psoriatic disease in selected patients. Upadacitinib mainly covers downstream gaps of multiple pathways: ① It inhibits JAK1/JAK2, blocking the IL-6/JAK2/STAT3 and TNF-α/JAK1 pathways (8), improving non-IL-23-dependent osteoarticular inflammation, especially targeting TNF-α-mediated cartilage degradation that cannot be affected by guselkumab. ②Guselkumab cannot inhibit IL-23 100%, small amount of residual IL-17 induce inflammation via JAK/STAT, which can be blocked by upadacitinib, explaining the rapid PASI reduction at 4 weeks (8). ③ Indirectly downregulates NF-κB activity, reducing complement-mediated neutrophil infiltration (3), further improving palmoplantar pustules.

The current case achieved successful outcomes with the combination of guselkumab and upadacitinib. No osteoarticular tenderness was observed 2 weeks after treatment initiation, and cutaneous symptoms improved significantly at 4 weeks, with no recurrence noted within 6 months of treatment. Occasional localized eruptions occurred, which were confirmed as Malassezia folliculitis with positive spores by laboratory examination, and resolved with topical medication. No other significant adverse effects were reported.

## Data Availability

The original contributions presented in the study are included in the article/supplementary material. Further inquiries can be directed to the corresponding authors.
